# Diverse Regulation of Vitamin D Receptor Gene Expression by 1,25-Dihydroxyvitamin D and ATRA in Murine and Human Blood Cells at Early Stages of Their Differentiation

**DOI:** 10.3390/ijms18061323

**Published:** 2017-06-21

**Authors:** Sylwia Janik, Urszula Nowak, Agnieszka Łaszkiewicz, Anastasiia Satyr, Michał Majkowski, Aleksandra Marchwicka, Łukasz Śnieżewski, Klaudia Berkowska, Marian Gabryś, Małgorzata Cebrat, Ewa Marcinkowska

**Affiliations:** 1 Laboratory of Molecular and Cellular Immunology, Department of Tumor Immunology, Institute of Immunology and Experimental Therapy, Polish Academy of Science, Weigla 12, 53-114 Wrocław, Poland; sw90@interia.pl (S.J.); bijbo@interia.pl (A.Ł.); michal.majkowski@iitd.pan.wroc.pl (M.M.); lukasz.sniezewski@iitd.pan.wroc.pl (Ł.Ś.); cebrat@iitd.pan.wroc.pl (M.C.); 2 Laboratory of Protein Biochemistry, Faculty of Biotechnology, University of Wrocław, Joliot-Curie 14a, 50-383 Wrocław, Poland; urszula.nowak.bio@gmail.com (U.N.); anastasiia.satyr@gmail.com (A.S.); alexandramarchwicka@interia.pl (A.M.); airealphiel@wp.pl (K.B.); 3 First Department of Obstetrics and Gynecology, Wrocław Medical University, Chałubińskiego 3, 50-368 Wrocław, Poland; mst_gabrys@post.pop.pl

**Keywords:** blood cells, vitamin D receptor, retinoic acid receptors, expression, *CYP24A1*, *CYP26A1*, differentiation, hematopoietic stem cells

## Abstract

Vitamin D receptor (VDR) is present in multiple blood cells, and the hormonal form of vitamin D, 1,25-dihydroxyvitamin D (1,25D) is essential for the proper functioning of the immune system. The role of retinoic acid receptor α (RARα) in hematopoiesis is very important, as the fusion of RARα gene with PML gene initiates acute promyelocytic leukemia where differentiation of the myeloid lineage is blocked, followed by an uncontrolled proliferation of leukemic blasts. RARα takes part in regulation of *VDR* transcription, and unliganded RARα acts as a transcriptional repressor to *VDR* gene in acute myeloid leukemia (AML) cells. This is why we decided to examine the effects of the combination of 1,25D and all-*trans*-retinoic acid (ATRA) on *VDR* gene expression in normal human and murine blood cells at various steps of their development. We tested the expression of *VDR* and regulation of this gene in response to 1,25D or ATRA, as well as transcriptional activities of nuclear receptors VDR and RARs in human and murine blood cells. We discovered that regulation of *VDR* expression in humans is different from in mice. In human blood cells at early stages of their differentiation ATRA, but not 1,25D, upregulates the expression of *VDR*. In contrast, in murine blood cells 1,25D, but not ATRA, upregulates the expression of *VDR*. VDR and RAR receptors are present and transcriptionally active in blood cells of both species, especially at early steps of blood development.

## 1. Introduction

Retinoic acid (RA), an active metabolite of vitamin A, and the hormonal form of vitamin D, 1,25-dihydroxyvitamin D (1,25D), are very active compounds, which regulate many important cellular processes, such as differentiation and proliferation [1,2]. RA and 1,25D are the ligands for nuclear receptors, which act as transcription factors after binding the ligand. A dominating RA metabolite in humans is all-*trans*-RA (ATRA), which binds with high affinity to all retinoic acid receptors (RARα, β and γ). A less abundant metabolite, which is nevertheless present in almost all tissues, is 13-*cis*-RA, which most probably serves as a depot for isomerisation to ATRA or to 9-*cis*-RA. 9-*cis*-RA is hard to detect in human tissues, and it binds predominantly to retinoid X receptors (RXRα, β and γ) [3,4]. 1,25D is a ligand to only one vitamin D receptor (VDR), which in its active state forms heterodimers with RXRs [5]. After ligation, VDR and RARs undergo conformational changes that induce binding to specific sequences in the promoter regions of target genes. These sequences are called vitamin D response elements (VDRE) and retinoic acid response elements (RARE) [6]. The role of RARα in blood development is very important, as the fusion of the RARα gene with the PML gene, caused by a translocation t(15;17), initiates acute promyelocytic leukemia where differentiation of the myeloid lineage is blocked at a promyelocyte stage and followed by an uncontrolled proliferation of leukemic blasts [7]. It is also well known and widely accepted that VDR is present in multiple blood cells, and that the correct levels of 1,25D are essential for proper functioning of the immune system [8]. Both compounds, ATRA and 1,25D, can be used in therapy to induce differentiation of acute myeloid leukemia (AML) blasts. ATRA induces differentiation of these blasts to granulocyte-like cells, and 1,25D to monocyte-like cells [9,10]. Past research indicated a synergistic cancer differentiation effect by using a combination of 1,25D and ATRA [11]. However, our recent experiments have revealed that in AML cell lines the effect of combination treatment varies, due to either down- or upregulation of *VDR* expression in response to ATRA, depending on the AML cell line examined [12,13].

Since beneficial effects of 1,25D and ATRA combination treatment in anticancer therapy have been reported and their wider use postulated [14], the effects of such combination towards normal cells should be addressed. Hematopoiesis seems to be the most relevant process which might be influenced by ATRA and 1,25D. The roles of vitamin A and its most active metabolites during hematopoiesis have been extensively studied and are well appreciated [15]. The actions of RA are multiple, and they start as early as in embryonal yolk sac and aorta-gonad-mesonephros, where RA causes the appearance of hematopoietic progenitors from the hemogenic endothelium [16]. In adult hematopoiesis, RA is important for granulopoiesis, and it controls differentiation of B and T lymphocytes [15]. However, it should be remembered that, due to difficulties in the use of human models of hematopoiesis, mice models have often been used in the experiments [15]. The role of 1,25D in hematopoiesis is less well documented than that of ATRA; moreover, some of the data come from zebrafish models. It should be remembered that, in contrast to humans and mice, there are two forms of VDR in zebrafish [17]. However, the available data show that the correct levels of 1,25D are necessary to maintain hematopoietic stem and progenitor cells (HSPCs) [18]. It was also shown that in human hematopoietic stem cells (HSCs) exposed to physiological concentrations of 1,25D, markers of monocytic differentiation are induced [19].

The gene encoding human VDR is located on chromosome 12. This gene is composed of 14 exons, and translation of VDR protein starts from the exon 2. Region 5′ of human *VDR* gene is very complex, and is composed of the seven exons 1a–g. These exons, together with corresponding promoter regions, are alternatively used for *VDR* transcription in different tissues. Transcripts starting from exon 1a and from exon 1d are regulated by the common promoter upstream to exon 1a, and the exons 1f and 1c have separate upstream promoters [20]. Our recent experiments have revealed a new exon, 1g, regulated from the promoter of exon 1a. Exon 1g is used in *VDR* transcripts present in AML cells [13]. Multiple publications confirm that *VDR* expression in humans is regulated in response to ATRA [12,21,22,23], while there are conflicting reports concerning regulation of human *VDR* by 1,25D [13,24,25,26].

The murine *VDR* gene is located on chromosome 15, and its composition is less complex than in humans. In the 5’ UTR region of *VDR* gene, exons 1 and 2 were identified, which show strong homology to human 1a and 1c, respectively [27]. Although exon 1d is well conserved (1d-like), transcripts containing this exon have not been reported in mice. The sequence similarity of the exons 1f and 1b is low between man and mice. Translation of mouse VDR protein starts from exon 3 [28]. It has been shown that transcription of *VDR* is upregulated in response to 1,25D in murine osteoblasts [29,30].

This is why we decided to examine the effects of the 1,25D and ATRA combination on *VDR* gene expression in blood cells at various steps of their development. We were interested in discovering if, in normal human blood cells, transcriptional variants of the *VDR* gene are as multiple as in AML cells, and if they are regulated in response to ATRA and 1,25D. Since the availability of human hematopoietic cells for experiments is very limited, we decided to examine whether human cells could be replaced in this type of studies with murine blood model.

An important question in studies concerning nuclear receptors is whether or not they are transcriptionally active in the cells. It is therefore important to be able to study expression of the genes that are specific targets of regulation by either VDR or RARs. In the case of VDR, expression of the gene which encodes 24-hydroxylase of 1,25D (*CYP24A1*) is the best measure of VDR’s activity. 24-Hydroxylase of 1,25D is the central enzyme in the catabolism of 1,25D to calcitroic acid. *CYP24A1* is the most strongly regulated out of all 1,25D-target genes [31]. This is why 1,25D-dependent upregulation of *CYP24A1* confirms that VDR protein is expressed and active in cells. *CYP24A1* is upregulated in response to 1,25D, but not in response to ATRA [13], and its expression can also be detected in human HSCs [18]. We decided to search for a similar sensor of RARs transcriptional activity. The effective concentration of the most active metabolite of vitamin A, ATRA is strictly controlled in the human body. In order to maintain safe concentrations of ATRA, its catabolism is also strictly regulated [32]. The major catabolizing enzyme is retinoic acid 4-hydroxylase (CYP26A1), whose transcription is upregulated in response to ATRA [33] by activating RARE, which is present in the *CYP26A1* promoter region [34]. Thus, our first aim was to verify that *CYP26A1* is regulated by ATRA in RAR-positive cells, and that it is not regulated by 1,25D in VDR-positive cells.

## 2. Results

### 2.1. Activation Of Retinoic Acid 4-Hydroxylase (CYP26A1) Gene Expression in Acute Myeloid Leukemia (AML) Cells

**Figure 1:**
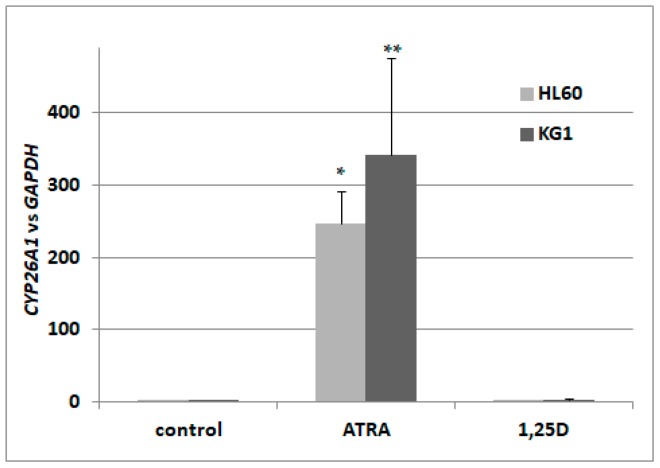
Expression of retinoic acid 4-hydroxylase gene (*CYP26A1*) in acute myeloid leukemia (AML) cell lines exposed to all-*trans*-retinoic acid (ATRA) or to 1,25-dihydroxyvitamin D (1,25D). HL60 and KG1 cells were exposed to 1 μM ATRA or to 10 nM 1,25D and after 96 h the expression of *CYP26A1* mRNA was measured by Real-time polymerase chain reaction (PCR). The bars represent mean values (±standard error of the mean (SEM)) of the fold changes in mRNA levels relative to glyceraldehyde 3-phosphate dehydrogenase (*GAPDH*) mRNA levels. Values significantly different from these obtained for respective control cells are marked with asterisks (* *p* < 0.01, ** *p* < 0.05).

In a search for the sensor of transcriptional activity of RARs, we decided to concentrate on isoforms of CYP26. According to the available literature, CYP26A1 is the most ubiquitously expressed isoform [32], present in HSCs [35], and exhibits the greatest increase upon stimulation with ATRA [36]. Therefore, we wanted to verify that the gene encoding CYP26A1 is upregulated in AML cells in response to ATRA-treatment, and what the kinetics of its activation are. Our initial experiments conducted on HL60 cells revealed that the kinetics of *CYP26A1* upregulation are: a slow, significant increase can be seen after 72 h of treatment, and after 96 h, expression is about 200–250 times higher than in vehicle-treated cells. Then we treated HL60 cells with ATRA at concentrations ranging from 10 nM to 1 μM for 96 h. It appeared that at 100 nM we could detect noticeable upregulation of *CYP26A1* expression, but in order to obtain significant upregulation, cells had to be exposed to 1 μM ATRA. In our next experiments, HL60 cells were exposed to either 1 μM ATRA or to 10 nM 1,25D. From our past experiments, we know that 10 nM 1,25D significantly upregulates the gene encoding CYP24A1, and that ATRA does not regulate this gene in many different AML cell lines [13]. Now we wanted to determine if 1,25D has any influence on *CYP26A1* expression. HL60 cells are very sensitive to 1,25D [12], and are moderately sensitive to ATRA. We also tested how *CYP26A1* is regulated in KG1 cells. These cells have higher expression levels of *RARA* and *RARB* genes, and a higher level of RARα protein than HL60 [13,37], but they have low expression of *VDR* [13]. Our experiments showed that the expression of *CYP26A1* could be upregulated in HL60 cells by ATRA by up to 250 times, compared to the control cells, but is not regulated by 1,25D. In KG1 cells, ATRA induced upregulation of *CYP26A1* as well, and again this gene was not regulated by 1,25D (Figure 1). Therefore we decided to measure levels of *CYP26A1* mRNA in order to detect transcriptional responses to ATRA in our next experiments performed using blood cells.

### 2.2. Detection of Transcriptional Variants of Vitamin D Receptor Gene (VDR) and Their Regulation in Response to All-trans-Retinoic Acid (ATRA) in Normal Human Blood Cells

**Figure A1:**
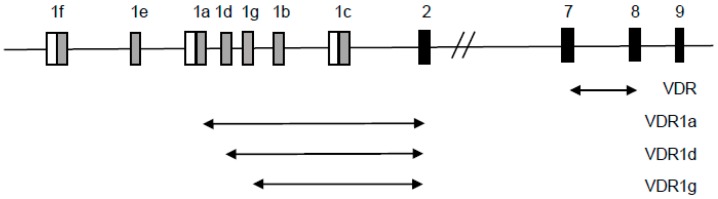
Graphical representation of transcriptional variants of human *VDR* detected in Real-time PCR. Primer sequences are given in Materials and Methods. All *VDR* transcripts were detected using primers which amplify product on the border of exons 7 and 8 (VDR). Transcripts that start at exon 1a were detected using primers which amplify product on the border of exons 1a and 2 (*VDR1a*). Transcripts which start at exon 1d were detected using primers which amplify product on the border of exons 1d and 2 (*VDR1d*) and transcripts which start at exon 1g were detected using primers which amplify product on the border of exons 1g and 2 (*VDR1g*). Black boxes represent coding exons, gray boxes represent noncoding exons and white boxes represent defined promoter regions.

In our previous experiments, we found that in HL60 cells *VDR* transcripts originated from exon 1a, which was spliced to exon 2. KG1 cells express a more varied set of *VDR* transcripts than HL60 cells. In unstimulated KG1 cells, transcript variants originating from exon 1a, 1d and 1g were detected. In these cells, transcripts containing exon 1b present in transcripts originating from exon 1a or 1g were also found. Transcripts originating from exons 1a and 1g appeared to be regulated by ATRA [13]. Now we wanted to verify whether, in normal human blood cells, such a large variety of *VDR* transcripts could also be detected. For this reason, we used variant-specific primers in order to detect the above transcripts and to check if they are regulated in response to ATRA. Primer sequences for general *VDR* transcripts and for variant-specific transcripts are given in the Materials and Methods section, and graphical representation of polymerase chain reaction (PCR) products is shown in Figure A1.

**Figure 2:**
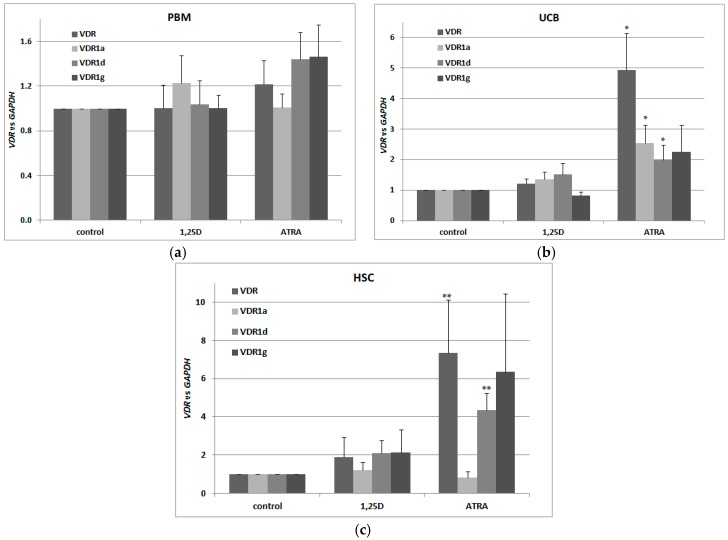
Regulation of *VDR* by 1,25D or ATRA in human blood cells. Human blood cells were isolated as described in Materials and Methods. The cells were exposed ex vivo to 10 nM 1,25D or to 1 μM ATRA for 96 h. Then mRNA was isolated and expression of *VDR* transcriptional variants was measured by Real-time PCR for peripheral blood of healthy adults (PBM) (**a**), mononuclear cells from human umbilical cord blood (UCB) (**b**) and hematopoietic stem cells (HSC) (**c**). The bars represent mean values (±SEM) of the fold changes in mRNA levels relative to *GAPDH* mRNA levels. Values that differ significantly from these obtained for respective control are marked with asterisks (* *p* < 0.01, ** *p* < 0.05).

**Figure A2:**
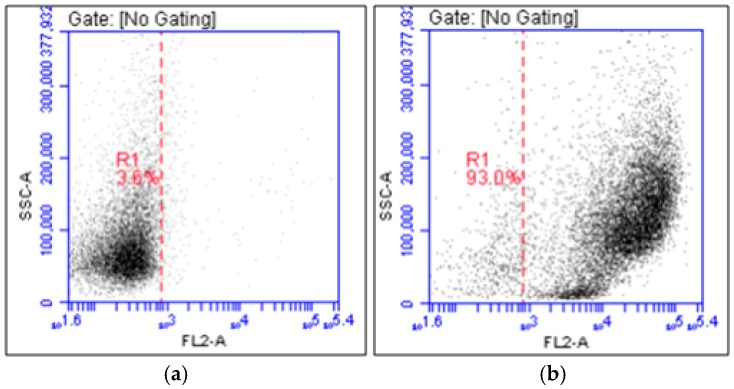
Human CD34+ cells were isolated from UCB as described in Materials and Methods. The cells before sorting, and the cells after magnetic sorting were stained using CD34-PE antibody. Cell fluorescence was detected in flow cytometry using Accuri flow cytometer (Becton Dickinson). Red line separates CD34-negative cells (left side of red line) from CD34-positive cells (right side of red line). Sample staining is presented for unsorted (**a**) and sorted (**b**) cells.

For our experiments, we used three different sets of blood cells. The first set consisted of the mononuclear cells from the peripheral blood of healthy adults (PBM), isolated using Histopaque-based centrifugation. The second (UCB) consisted of mononuclear cells from human umbilical cord blood, which is strongly enriched in HSCs and progenitor cells in comparison to peripheral blood [38]. The third set (HSCs) was up to 95% enriched in CD34+ cells isolated from UCB (efficacy of CD34+ cells isolation is presented in Figure A2). All three transcriptional variants were present in human blood cells, however only in UCB and HSC cells were *VDR* and its transcriptional variants regulated by ATRA (Figure 2a–c). Our results suggest that there also exist other transcriptional variants of *VDR* in addition to the tested ones. This is because, the regulation of total *VDR* transcripts in response to ATRA is stronger than the regulation of specific transcripts identified in our earlier studies.

### 2.3. Detection Of Transcriptional Activities of VDR and Retinoic Acid Receptors (RARs) in Normal Human Blood Cells

**Figure 3:**
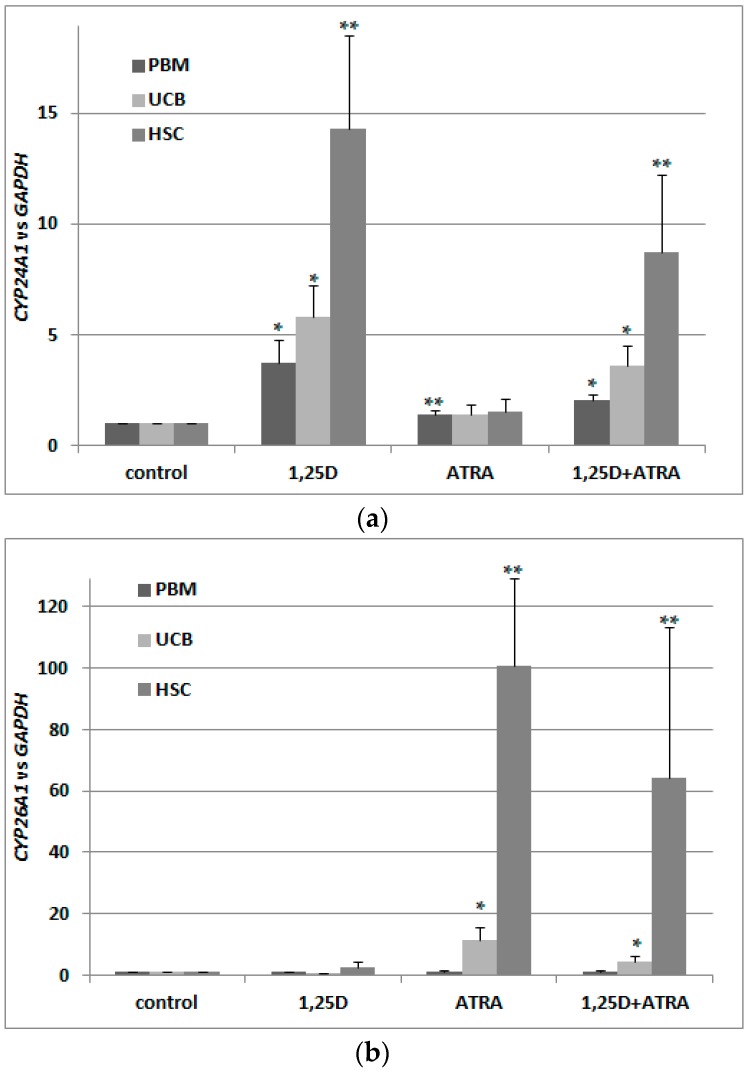
Transcriptional activity of VDR (**a**) and RARs (**b**) in human blood cells. Human blood cells were isolated as described in Materials and Methods. The cells were exposed ex vivo to 10 nM 1,25D ± 1 μM ATRA for 96 h. Then mRNA was isolated and *CYP24A1* (**a**) or *CYP26A1* (**b**) expression was measured by Real-time PCR. The bars represent mean values (±SEM) of the fold changes in mRNA levels relative to *GAPDH* mRNA levels. Values that are significantly higher than from these obtained for respective control are marked with asterisks (* *p* < 0.01, ** *p* < 0.05).

Next, we wanted to verify whether *VDR* transcripts in these cells are translated into a transcriptionally active VDR protein. This is why we exposed the blood cells to 10 nM 1,25D and/or 1 μM ATRA and tested expression of *CYP24A1* in these cells. As presented in Figure 3a, 1,25D caused an upregulation of *CYP24A1* mRNA levels, but co-treatment with 1 μM ATRA did not cause further upregulation. This shows that ATRA-induced upregulation of *VDR* transcription was not followed by an increase in transcriptionally active VDR protein. Then, we wanted to verify that ATRA did, indeed, activate RARs in these cells. This is why we checked the expression of *CYP26A1* in blood cells exposed to 1 μM ATRA for 96 h. As presented in Figure 3b, transcriptional activity in response to ATRA was very high in HSCs, and moderate in UCB cells. In PBM cells from healthy adults, ATRA did not cause upregulation of *CYP26A1*, which might indicate that RAR receptors are not present in these cells. This might also explain the lack of regulation of *VDR* in response to ATRA in PBM cells presented in Figure 2a. *CYP26A1* in normal blood cells was not upregulated in response to 1,25D (Figure 3b).

### 2.4. Detection of Transcriptional Variants of VDR in Murine Blood Cells Using 5′-Rapid Amplification of cDNA Ends (RACE) Assay

**Figure 4:**
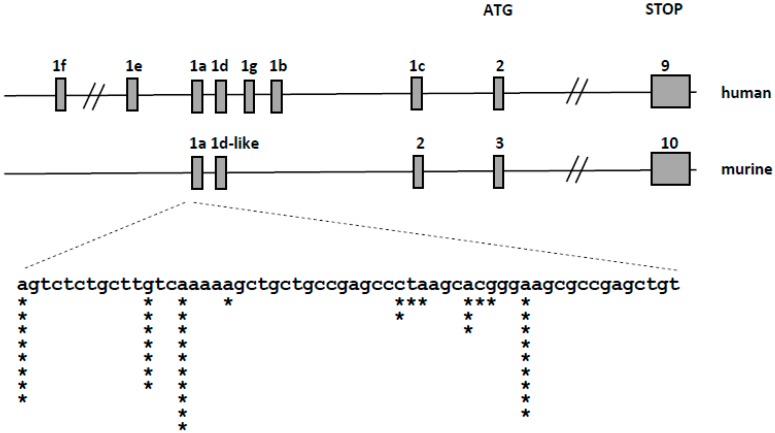
Organization of the human (**upper panel**) and murine (**middle panel**) *VDR* locus. Gray boxes represent exons. The expanded view (**bottom panel**) represents the genomic sequence surrounding the transcription start sites of the murine transcripts—asterisks represent the localization of the start sites identified during 5′-RACE, the number of asterisks—the number of transcripts starting at a given site.

The murine *VDR* transcript consists of 10 exons, of which exons 3–10 contain the open reading frame. The organization of the murine *VDR* locus is similar to human, however less non-coding 5′ exons has been identified in the murine locus (Figure 4 top). We used the 5’-RACE method to identify the 5′-ends and transcriptional start sites of *VDR* transcripts in the selected murine tissues: intestine, kidney and bone marrow. We have found that the transcription start sites of murine *VDR* are localized in a single region spanning 45 nucleotides, and identified only one splice variant of the *VDR* transcript (Figure 4 bottom). We did not find any strong tissue-specific preference in the transcription start sites used. This observation strongly suggests that, in contrast to the complex regulation of *VDR* transcription in human cells controlled by several promoters, *VDR* transcription in mice is controlled by a single promoter region.

### 2.5. Basal Expression of VDR in Murine Kidneys and in Blood Cells

**Figure 5:**
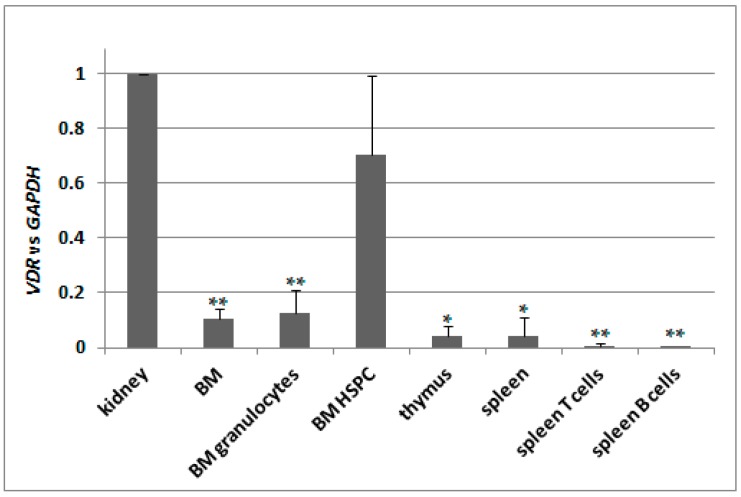
Expression of *VDR* in C57BL/6 mice. Tissues from 2 to 9 mice were isolated as described in Materials and Methods. mRNA was isolated and *VDR* expression was measured by Real-time PCR. The bars represent mean values (±SEM) of the fold changes in mRNA levels relative to *GAPDH* mRNA levels. Values that are significantly different from these obtained for kidney are marked with asterisks (* *p* < 0.01, ** *p* < 0.05).

Knowing that there is only one transcriptional variant of *VDR* gene present in C57BL/6 mice, we decided to test its basic expression in different tissues. We used kidneys as a reference 1,25D-responsive organ, therefore we normalized expression of *VDR* in remaining tissues to the expression measured for kidneys. We used pooled bone marrow cells (BM), bone marrow granulocytes isolated using CD45/SSC-based sorting (BM granulocytes), hematopoeitic stem and progenitor cells isolated from bone marrow (BM HSPC), pooled thymocytes isolated from thymus, pooled spleen cells, spleen T cells isolated using anti-CD3 and spleen B cells isolated using anti-CD19 antibody. Indeed, the expression of *VDR* appeared to be the highest in kidneys, and in all other tissues tested it was significantly lower except of BM HSPC. The results are presented in Figure 5.

### 2.6. Regulation of VDR Expression by 1,25D and ATRA in Murine Kidneys and Blood Cells

**Figure 6:**
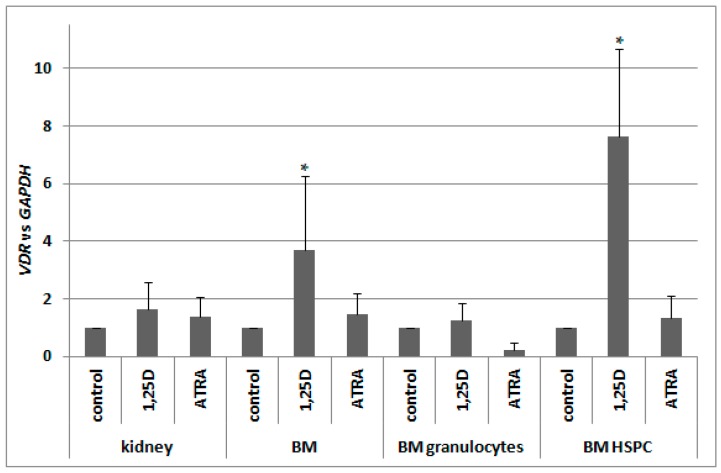
Regulation of *VDR* by 1,25D or ATRA in murine cells. Tissues from 3 to 10 mice were isolated as described in Materials and Methods. The cells were exposed ex vivo to 10 nM 1,25D or to 1 μM ATRA for 96 h. Then mRNA was isolated and *VDR* expression was measured by Real-time PCR. The bars represent mean values (±SEM) of the fold changes in mRNA levels relative to *GAPDH* mRNA levels. Values that differ significantly (*p* < 0.01) from those obtained for respective controls are marked with asterisks.

Our next goal was to verify whether, in murine blood cells, *VDR* expression is regulated by ATRA in a similar manner to human blood cells. Since previous reports indicated a 1,25D-induced regulation of *VDR* in murine osteoblasts [29,30], we decided to also test this compound. Therefore we took different fractions of cells from bone marrow, and kidney cells as a control of 1,25D-responsive tissue, and exposed them ex vivo to either 10 nM 1,25D or to 1 μM ATRA for 96 h, and then we measured the levels of *VDR* mRNA relative to glyceraldehyde 3-phosphate dehydrogenase (*GAPDH*) expression. In kidneys, expression of *VDR* was not regulated to a significant degree by either of the compounds used. In pooled BM cells, there was significant upregulation of *VDR* expression in response to 1,25D, which was not present in BM granulocytes. The strongest and the most significant upregulation of *VDR* expression in response to 1,25D was detected in BM HSPC. ATRA did not regulate *VDR* expression in all of the cells tested (Figure 6).

### 2.7. Detection of Transcriptional Activities of RARs and VDR in Murine Blood Cells

**Figure 7:**
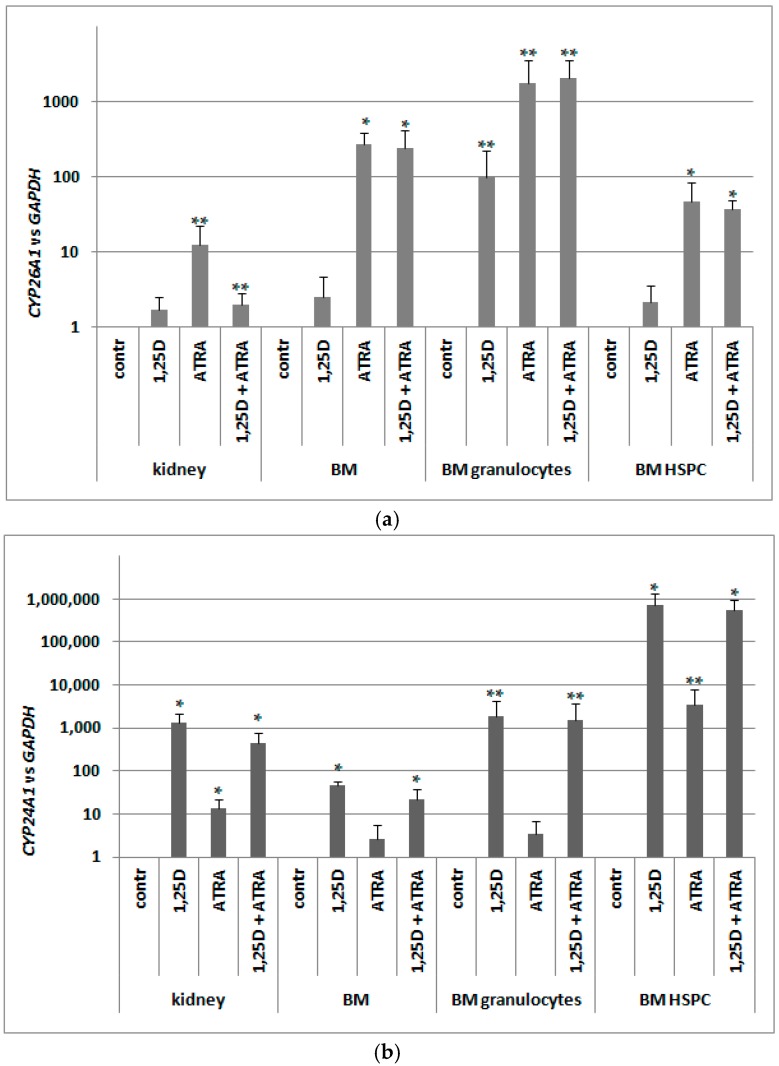
Transcriptional activity of RARs (**a**) and VDR (**b**) in murine cells. Tissues from 3 to 8 mice were isolated as described in Materials and Methods. The cells were exposed ex vivo to 10 nM 1,25D ± 1 μM ATRA for 96 h. Then mRNA was isolated and *CYP26A1* (**a**) or *CYP24A1* (**b**) expression was measured by Real-time PCR. The bars represent mean values (±SEM) of the fold changes in mRNA levels relative to *GAPDH* mRNA levels. Values that are significantly higher than these obtained for respective control are marked with asterisks (* *p* < 0.01, ** *p* < 0.05).

Since in murine blood cells an expression of *VDR* was not regulated by ATRA as in human blood cells, we hypothesized that RARs might not be present in these cells. Since RARs are ligand-activated transcription factors, and ATRA is a universal ligand for all RAR isoforms, we decided to test expression of *CYP26A1* as a RAR-target gene. Again, we took different populations of cells from bone marrow and kidney cells, and exposed them ex vivo to either 10 nM 1,25D or/and to 1 μM ATRA for 96 h. After ex vivo stimulation, mRNA was isolated from the cells, the expression of *CYP26A1* was tested, and *GAPDH* expression was used as a reference. Relative expression of *CYP26A1* in control cells (exposed to vehicle) was calculated as 1. As presented in Figure 7a, *CYP26A1* was upregulated in all samples exposed to ATRA; the highest and most significant upregulation was observed in pooled BM cells and in BM HSPC. These results indicate that RARs are expressed in BM and specifically in HSPC, and are transcriptionally active.

Our next goal was to verify whether the *VDR* mRNA detected in murine blood cells was effectively translated into a transcriptionally active protein. Similarly as with human cells, we decided to test this activity by measuring the expression of its most strongly regulated target gene in the cells exposed to 10 nM 1,25D. As we used the cDNA obtained for the experiment described above, we tested expression of *CYP24A1* in the cells exposed ex vivo to either 10 nM 1,25D or/and to 1 μM ATRA for 96 h. Again *GAPDH* was used as a reference gene, and relative expression of *CYP24A1* in control cells (exposed to vehicle) was calculated as 1. As presented in Figure 7b, 1,25D-induced upregulation of *CYP24A1* is very high (more than 10^3^ times) and significant in kidney cells. However, 1,25D-induced expression of *CYP24A1* in BM HSPC is even higher, reaching a level of almost 10^6^ times that of vehicle-treated cells. This shows that in HSPCs exposed to 1,25D, *VDR* is present and is transcriptionally active. An unexpected result was obtained in kidney cells and in BM HSPCs exposed to ATRA. In these cells, we observed significant increase of *CYP24A1* expression. The molecular mechanism of this effect will be studied in our future experiments.

## 3. Discussion

The findings from studies of leukemia cell lines support the use of 1,25D as an anticancer agent, since 1,25D causes the growth arrest and differentiation of a wide variety of AML cell lines [39]. Our results revealed that there are patients whose AML blasts respond to 1,25D analogs with differentiation, while the blasts of others are resistant [39,40]. The possible reason of these differences may lie in the expression level of the *VDR* gene and the VDR protein level in AML cells. We have recently found that low *VDR* expression levels may be upregulated using ATRA and that unliganded RARα acts as transcriptional repressor to *VDR* [13]. The possibility of using RA analogs to induce differentiation of blasts was investigated for many years and in the case of ATRA it was successfully introduced into clinics to treat one subtype of AML [41]. Unfortunately, in other subtypes of leukemia, ATRA is not effective. This is why the combined use of 1,25D and ATRA, or a combination of their more active analogs, was postulated [14]. In AML cells, the *VDR* gene is transcribed in multiple variants, and some of them are transcriptionally regulated by ATRA. We thus wanted to ascertain whether similar transcriptional variants of VDR can be found in normal human blood cells. Our experiments revealed that transcripts *VDR1a*, *VDR1d* and *VDR1g* can also be detected in normal blood cells, however they are upregulated in response to ATRA, in a manner similar to KG1 cell line, only in blood cells found in UCB. *VDR* protein in UCB cells is transcriptionally active after exposure to 1,25D, and in PBM cells this transcriptional activity is much lower. Moreover, in contrast to AML cell lines, upregulation of *VDR* expression is not followed by an increased transcriptional activity of VDR protein. This might suggest that possible side effects of combination treatment using 1,25D and ATRA in normal human blood cells do not synergize.

It is widely accepted that the eventual cell fate during hematopoiesis is governed by spatiotemporal fluctuations in transcription factor concentrations, which either cooperate or compete in driving target gene expression [42]. Nuclear receptors for vitamin A and vitamin D do not belong to the set of the most important hematopoietic regulators, but their roles in blood cell differentiation are becoming apparent and appreciated. The availability of human HSC cells is limited, and their differentiation can’t be studied in vivo. This is why the majority of the available data about blood cell formation comes from murine models, however one should be aware that human hematopoiesis does not reflect murine hematopoiesis in all aspects [43]. This might also concern the roles of VDR and RARs in blood cell formation. As mentioned in the Introduction, the organization and regulation of the *VDR* locus in humans and mice are different, and the 5′ UTR region composition is less complex in mice. Despite the high resemblance of the symptoms of *VDR* knock-out in mice and inactivating mutations of *VDR* in humans, not all findings concerning vitamin D endocrine systems in mice are present in humans [44]. This is why we addressed regulation of *VDR* transcription in murine blood cells in parallel to human cells.

Our results indicate that there are differences in the regulation of *VDR* transcription between mice and man. In contrast to human cells, in murine cells ATRA does not influence *VDR* expression, even though RARs are present and transcriptionally active. On the other hand, the *VDR* gene in murine blood is positively auto-regulated by the VDR ligand, 1,25D. This positive feedback causes transcriptional activity of VDR to be particularly high in murine HSPC exposed to 1,25D. This might indicate that VDR is important for murine blood cells at early stages of their commitment. Such auto-regulation of VDR does not occur in human blood cells. Since there is no good alternative for testing the toxicity of potential drugs other than rodents, we suggest that observations concerning toxic effects of 1,25D in mice should be translated to humans with caution.

Another issue to consider is the fact that there are differences in the vitamin D system between mice and man. 1,25D is a steroid hormone, which is entirely produced by organisms from 7-dehydrocholesterol, and biologically activated by subsequent hydroxylations at carbons C25 and C1 [45]. This requires exposure to the UVB light, and vitamin D must only be delivered with food for people who live in regions deficient in sunlight. Mice are much less likely to produce vitamin D in high amounts from exposure to sunlight due to their fur, as well as their nocturnal and underground activities. Therefore, the role and regulation of *VDR* is likely to be different in these two distinct species, and should be taken into consideration when vitamin D compounds are being tested in mice. Very high positive auto-regulation of *VDR* expression in murine blood cells at their early steps of development, which does not occur in humans, might cause unwanted side-effects of 1,25D, or of its highly active analogues, to be more pronounced in mice than in humans.

## 4. Materials and Methods

### 4.1. Chemicals

1,25D was purchased from Cayman Europe (Tallinn, Estonia) and ATRA was from Sigma-Aldrich (St. Louis, MO, USA). The compounds were dissolved in an ethanol to reach 1000× final concentrations, and subsequently diluted in the culture medium to the concentration required for experiments.

### 4.2. Cell Lines and Normal Cells

HL60 cells were acquired from the cell bank at the Institute of Immunology and Experimental Therapy in Wrocław, Poland and KG1 cells were purchased from the German Resource Center for Biological Material (DSMZ GmbH, Braunschweig, Germany). The cells were cultured in RPMI-1640 medium (Biowest, Nuaillé, France) with 10% fetal bovine serum (FBS), 2 mM l-glutamine, 100 units/mL penicillin and 100 µg/mL streptomycin (all from Sigma-Aldrich) and maintained at standard cell culture conditions.

Human UCB was obtained post-delivery at the First Department of Obstetrics and Gynecology, Wrocław Medical University (Wrocław, Poland) from mothers who gave informed consent for this study. The study was accepted by the local Ethical Committee. Two to eight mL of cord blood were diluted with PBS in 1:1 ratio. Diluted blood was carefully layered onto the equal volume of Histopaque 1077 (Sigma-Aldrich), and centrifuged at 400× *g* for 30 min. The opaque interface containing mononuclear cells was moved to fresh sterile tube, and washed three times with PBS. The cells were transferred to Biotarget-1 (Biological Industries, Kibbutz Beit-Haemek, Israel) medium containing 4 mM l-glutamine, 100 units/mL penicillin and 100 µg/mL streptomycin and maintained at standard cell culture conditions.

The experiments using animals were performed according to the procedures approved by the First Local Ethical Commission for Animal Experimentation in Wrocław at the Institute of Immunology and Experimental Therapy (permit numbers 21/2016/W, 21/2016/U, 20/2016/U issued on 5 January 2016). Cell suspensions from 8 week old C57BL/6 mice were prepared as follows: bone marrow cells were isolated by washing the femur and tibia with ice-cold PBS stream. Spleen and thymus were washed with ice-cold PBS and strained through 30-μm mesh. Kidneys were washed to remove blood, cut into small 1–2 mm^2^ pieces and incubated in 0.5 mL with colagenase type II (1 mg/mL) and DNAse I (10 units/mL) at 37 °C for 40 min. Cells isolated from bone marrow, spleen and thymus were treated with red blood cell lysis buffer (155 mM NH_4_Cl, 10 mM KHCO_3_, 0.1 mM ethylenediaminetetraacetic acid) to remove erythrocytes. All tissues and cells were mechanically dissociated by the syringe trituration, washed twice with PBS by centrifugation (400 rcf, 5 min, 4 °C) and resuspended in PBS supplemented with 5% FBS. Single cell suspension was filtered through 70-μm mesh.

### 4.3. Sorting of Blood Cells and Flow Cytometry

Human HSCs were sorted from cord blood mononuclear cells using the Miltenyi MACS CD34 Isolation Kit (Miltenyi Biotec, Bergisch Gladbach, Germany) in accordance with the manufacturer’s instructions. Briefly, cord blood mononuclears were resuspended in Separation Buffer (PBS with 10% bovine serum albumin (BSA)) and incubated with FcR Blocking Reagent and magnetic microbeads conjugated to monoclonal mouse anti-human CD34 antibody for 30 min at 4 °C. Labeled cell suspension was sorted using magnetic separator. After three washes with Rinsing Solution (PBS supplemented with 2 mM EDTA and 0.5% BSA), the column was removed from the separator and labeled CD34+ cells were eluted with 1 mL of Rinsing Solution. To determine the purity of CD34+ cell fraction, 1 × 10^5^ cells were stained with phycoerythrin (PE)-conjugated anti-CD34 (Becton Dickinson, San Jose, CA, USA) monoclonal antibody for 60 min on ice. Isotype-identical monoclonal antibodies served as controls. Next, the stained cells were analyzed using flow cytometry (BD Accuri™ C6, Becton Dickinson, San Jose, CA, USA). The purity ranged from 92% to 95%, and sample staining is presented in Figure A1. CD34+ cells were grown in Stemline Hematopoietic Stem Cell Expansion Medium with 4 mM l-glutamine, 100 units/mL penicillin and 100 µg/mL streptomycin, recombinant human cytokines (all from ImmunoTools, Friesoythe, Germany): stem cell factor (100 ng/mL), thrombopoietin (100 ng/mL) and granulocyte colony-stimulating factor (100 ng/mL).

Hematopoietic stem and progenitor cells were isolated from murine bone marrow using Mouse Hematopoeitic Progenitor Cell Isolation Kit (Stemcell, Cologne, Germany) according to the manufacturer’s recommendations. Briefly, the cells were resuspended in PBS (with 2% FBS and 1 mM EDTA, rat serum 50 μL/mL at the density of 1 × 10^8^ cells/mL) and incubated with EasySep Mouse Hematopoietic Progenitor Cell Isolation Cocktail (50 μL/mL) for 15 min at 4 °C. Next, EasySep^TM^ Streptavidin RapidSpheres (75 μL/mL) were added and after 10 min of incubation the cell suspension was sorted with magnets. The purity of the obtained population was monitored by flow cytometry (FACS-Calibur, Becton Dickinson, San Jose, CA, USA) using anti-c-kit-APC (eBioscience, Vienna, Austria) and anti-Sca-1-FITC (eBioscience) staining. According to the manufacturer, the lineage antigen-negative cell content of the isolated fraction typically ranges from 60% to 84%. In our experiments c-kit+ cells constituted 65% of sorted population. Stemline Hematopoietic Stem Cell Expansion Medium (Sigma-Aldrich) and recombinant murine cytokines (all from ImmunoTools): stem cell factor (50 ng/mL), Flt3-ligand (50 ng/mL), thrombopoietin (50 ng/mL) and interleukin-6 (10 ng/mL) were used for further ex vivo culture of the isolated cells.

**Figure A3:**
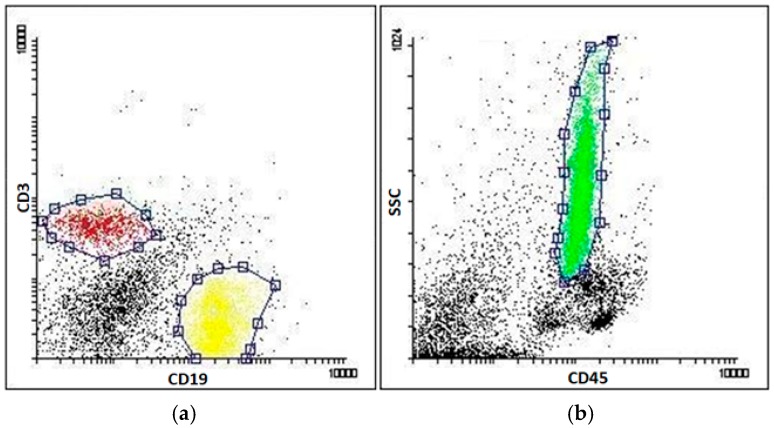
Murine BM leukocytes, T cells and B cells were sorted as described in Materials and Methods. (**a**) Murine spleen cells were stained with anti-CD3-APC and anti-CD19-PE antibodies to isolate mature T (red gate) and B (yellow gate) cells, respectively; (**b**) Bone marrow cells were stained with anti-CD45-FITC antibody to isolate granulocytes, using CD45/SSC-based sorting criteria (green gate). Cells were sorted using FACS-Aria (Becton Dickinson).

Murine spleen cells were stained with anti-CD3-APC and anti-CD19-PE antibodies (Becton Dickinson) to isolate mature T- and B-cells, respectively (Figure A3a). Bone marrow cells were stained with anti-CD45-FITC antibody (Becton Dickinson) to isolate granulocytes, using CD45/SSC-based sorting criteria (Figure A3b). Cells were stained in 0.5 mL PBS supplemented with 2% FBS using 1 μg of each antibody for 30 min on ice. Cells were sorted using FACS-Aria (Becton Dickinson).

### 4.4. 5′-RACE Assay

In order to identify the transcriptional start sites for murine *VDR* transcript(s), 5’-RACE was used [46]. Ten micrograms of total RNA were isolated from intestine, kidney or bone marrow of C57BL/6 mice and then processed as before: digested with calf alkaline phosphatase (CIP, New England Biolabs, Ipswich, MA, USA) in the presence of RiboLock RNAse inhibitor for 1 h at 37 °C and purified by extraction with TRI Reagent (Sigma-Aldrich). Half of the CIP-digested RNA was treated with tobacco acid pyrophosphatase (TAP, Epicentre, Madison, WI, USA) for 1 h at 37 °C in 10 μL reaction mixture containing TAP buffer, 0.5 units of TAP and 20 units of RiboLock RNAse inhibitor. 2 μL of TAP-treated RNA was ligated with a RNA oligonucleotide (5’-GCUGAUGGCGAUGAAUGAACACUGCGUUUGCUGGCUUUGAUGAAA-3′) for 1 h at 37 °C in a 10 μL reaction mixture containing 0.3 μg of the oligonucleotide, 5 U of RNA ligase (New England Biolabs), RNA ligase buffer and 20 U of RiboLock RNase inhibitor. 2 μL of RNA was then reverse transcribed using SuperScript III reverse transcriptase (Invitrogen, Carlsbad, CA, USA) and random hexamers. The cDNA was amplified in nested PCR reactions (2 × 20 cycles, annealing temp. 52 °C, in the presence of 1.2 M betaine) using primers complementary to 5′-adapter (5′-GCTGATGGCGATGAATGAACACTG-3′, 5′-CGCGGATCCGAACACTGCGTTTGCTGGCTTTGATG-3′) and exon 5 and 4 of *VDR* gene (5’-TCTGTGAGGATGAACTCCTTCATC-3′, 5′-TCCTTGGTGATGCGGCAATCTC-3′). The amplification products were directly cloned into pGEMT-easy vector. The individual plasmid clones were sequenced using SP6 primer (5’-ATTTAGGTGACACTATAG-3′) and BigDye 3.1 Terminator Cycle Sequencing Kit (Life Technologies, Carlsbad, CA, USA). The sequencing reaction was analyzed using ABI Prism 310 Genetic Analyzer (Applied Biosystems, Foster City, CA, USA). The obtained sequences of *VDR* transcripts were aligned with the genomic sequence of *VDR* gene using Spidey software (https://www.ncbi.nlm.nih.gov/spidey/ National Center for Biotechnology Information, Bethesda, MD, USA) to identify exons and transcriptional start sites.

### 4.5. cDNA Synthesis and Real-Time PCR

For PCR analyses, the cells were stimulated with 10 nM 1,25D and/or 1 μM ATRA for 96 h. RNA from unstimulated and stimulated cells was isolated using either TRI Reagent (Sigma-Aldrich), Extractme Total RNA Kit (DNA-Gdańsk, Gdańsk, Poland) (for >10^6^ cells) or PicoPure RNA Isolation Kit (ThermoFisher Scientific, Waltham, MA, USA) (for <10^6^ cells) according to manufacturer’s recommendations. Reverse transcription of 100 ng of total RNA (34 ng in case of human CD34+ cells) was done with High-Capacity cDNA Reverse Transcription Kit (ThermoFisher Scientific) using random hexamers. The Real-time PCR analysis was performed using Real-time PCR–PowerUp™ SYBR Green Master Mix (Applied Biosystems) or SensiFAST SYBR^®^ No-ROX Kit (Bioline, London, UK). For murine samples the reaction consisted of 40 cycles (95 °C for 15 s and 60 °C for 60 s), preceded by uracil-DNA glycosylase and AmpliTaq DNA polymerase activation at 50 °C for 120 s and 95 °C for 120 s, respectively, and was performed on BioRad CFX Connect apparatus (Bio-Rad Laboratories Inc., Hercules, CA, USA). The thermal profile for human samples consisted of 45 cycles (95 °C for 5 s, 54/58 °C for 10 s, 72 °C for 5 s) followed by one step at 95 °C for 2 min, the reaction was performed using CFX Real-time PCR System (Bio-Rad).

The following primer pairs were used:

*hGAPDH*: forward 5’-CATGAGAAGTATGACAACAGCCT-3′, reverse 5’-AGTCCTTCCACGATACCAAAGT-3′;

*hVDR*: forward 5’-CCTTCACCATGGACGACATG-3′, reverse 5’-CGGCTTTGGTCACGTCACT-3′;

*hVDR1a*: forward 5’-GCGGAACAGCTTGTCCACCC-3′, reverse 5’-GAAGTGCTGGCCGCCATTG-3′;

*hVDR1d*: forward 5’-GCTCAGAACTGCTGGAGTGG-3′, reverse 5’-GAAGTGCTGGCCGCCATTG-3′;

*hVDR1g*: forward 5’-TTGCTCATCCAGCTTCCCAGAC-3′, reverse 5’-GAAGTGCTGGCCGCCATTG-3′;

*hCYP24A1*: forward 5’-CTCATGCTAAATACCCAGGTG-3′, reverse 5’-TCGCTGGCAAAACGCGATGGG-3′;

*hCYP26A1*: forward 5’-CGCATCGAGCAGAACATTCG-3′, reverse 5’-GCTTTAGTGCCTGCATGT-3′;

*mGAPDH*: forward 5’-AACTTTGGCATTGTGGAAGG-3′, reverse 5’-ACACATTGGGGGTAGGAACA-3′;

*mVDR*: forward 5’-CACCTGGCTGATCTTGTCAGT-3′, reverse 5’-CTGGTCATCAGAGGTGAGGTC-3′;

*mCYP24A1*: forward 5’-CACGGTAGGCTGCTGAGATT-3′, reverse 5’-CCAGTCTTCGCAGTTGTCC-3′;

*mCYP26A1*: forward 5’-GCAGGCACTAAAACAATCGTC-3′, reverse 5’-GCTGTTCCAAAGTTTCCATGTC-3′. Relative quantification of gene expression was analyzed with the ΔΔ*C*_t_ method using *GAPDH* as the endogenous control.

### 4.6. Statistical Analysis

The sample distribution was assessed using the Shapiro-Wilk test. For samples with normal distribution, *t*-test was used to assess significance of differences. For the remaining samples, a non-parametric one-way ANOVA test followed by a Mann-Whitney *U* test was used for assessing the significance of the differences in gene expression levels.

## 5. Conlusions

The main findings of this paper indicate that there are differences in the regulation of *VDR* transcription between mice and man. Our study has revealed that in murine blood stem and progenitor cells expression of *VDR* is auto-regulated by ligand-activated VDR. Such auto-regulation does not occur in human blood stem and progenitor cells. On the contrary, in human cells expression of *VDR* is regulated by ligand-activated RARs, and this other kind of regulation does not occur in murine cells.

## Abbreviations

1,25D	1,25-Dihydroxyvitamin D
AML	Acute myeloid leukemia
ATRA	All-*trans*-retinoic acid
BM	Bone marrow
BSA	Bovine serum albumin
CYP24A1	24-Hydroxylase of 1,25D
CYP26A1	Retinoic acid 4-hydroxylase
FBS	Fetal bovine serum
HSC	Hematopoietic stem cells
HSPC	Hematopoeitic stem and progenitor cells
GAPDH	Glyceraldehyde 3-phosphate dehydrogenase
PCR	Polymerase chain reaction
PBM	Peripheral blood mononuclears
RA	Retinoic acid
RAR	Retinoic acid receptors
RARE	Retinoic acid response element
RXR	Retinoid X receptors
UCB	Umbilical cord blood
UTR	Untranslated region
VDR	Vitamin D receptor
VDRE	Vitamin D response element
